# A CRISPR-Cas9 System for Knock-out and Knock-in of High Molecular Weight DNA Enables Module-Swapping of the Pikromycin Synthase in its Native Host

**DOI:** 10.21203/rs.3.rs-6229288/v1

**Published:** 2025-03-27

**Authors:** Zhe-Chong Wang, Hayden Stegall, Takeshi Miyazawa, Adrian T. Keatinge-Clay

**Affiliations:** The University of Texas at Austin; The University of Texas at Austin; The University of Texas at Austin; The University of Texas at Austin

**Keywords:** pikromycin, type I PKSs, CRISPR/Cas9, riboswitch, updated module boundary, module-swapping, PKS engineering, S. venezuelae ATCC 15439

## Abstract

**Background:**

Engineers seeking to generate natural product analogs through altering modular polyketide synthases (PKSs) face significant challenges when genomically editing large stretches of DNA.

**Results:**

We describe a CRISPR-Cas9 system that was employed to reprogram the PKS in *Streptomyces venezuelae ATCC* 15439 that helps biosynthesize the macrolide antibiotic pikromycin. We first demonstrate its precise editing ability by generating strains that lack megasynthase genes *pikAI-pikAIV* or the entire pikromycin biosynthetic gene cluster but produce pikromycin upon complementation. We then employ it to replace 4.4-kb modules in the pikromycin synthase with those of other synthases to yield two new macrolide antibiotics with activities similar to pikromycin.

**Conclusion:**

Our gene-editing tool has enabled the efficient replacement of extensive and repetitive DNA regions within streptomycetes.

## BACKGROUND

The genus of bacteria, *Streptomyces,* is well known for its remarkable ability to produce diverse bioactive metabolites(1). Among these are the macrolide antibiotics, whose carbon skeletons are biosynthesized by modular polyketide synthases (PKSs)(2–4). These enzymatic assembly lines are comprised of modules, each of which minimally consists of three functional domains: an acyltransferase (AT) that selects an a-carboxyacyl extender unit, an acyl carrier protein (ACP) that fuses the extender unit to a polyketide intermediate bound to the upstream module, and a ketosynthase (KS) that acquires the acyl chain and fuses it with an extender unit bound to the downstream module. A module may also contain processing domains, such as a ketoreductase (KR), dehydratase (DH), and enoylreductase (ER), that modify the elongated polyketide intermediate(5–7). The traditional definition of a module, with KS at the upstream position, has guided module-swapping for three decades(8, 9). However, recent studies have revealed that processing domains evolutionarily comigrate with the downstream KS and that native ACP/KS interfaces should be preserved when engineering PKSs(10–15). Indeed, module-swapping using the updated module boundary, with KS at the downstream position, has been observed to consistently outperform module-swapping using the traditional boundary(14–16).

CRISPR-Cas9 (clustered regularly interspaced short palindromic repeats and CRISPR-associated protein 9) allows customizable edits to genomic DNA. It consists of a Cas9 endonuclease and a single-guide RNA (sgRNA). Cas9 navigates to the genomic locus specified by the sgRNA and generates a double-strand DNA break (DSB). This DSB can be imprecisely repaired by the non-homologous end-joining (NHEJ) (17). Alternatively, it can be precisely repaired through homologous recombination if a DNA donor template is available, as in homology directed repair (HDR). Since DSBs are cytotoxic and limit the efficiency of precise gene modification, researchers have developed various strategies including tuning Cas9 expression, base editing, and prime editing(18–20). Additionally, paired-guide prime editing techniques aim to facilitate the insertion of up to 5 kb(21, 22). Although these innovative approaches have made significant strides, current genome-editing methods still face challenges inserting or replacing large DNA segments at specific genome loci with high efficiency. Cas9 expression can be tuned using riboswitches, regulatory RNA elements found across a wide range of organisms(23, 24). Riboswitches undergo conformational changes upon binding to small molecule ligands and can regulate gene expression(25–27). Theophylline-responsive riboswitches can regulate gene expression in streptomyces and are valuable tools due to their high specificity, simple and reversible control, minimal genetic engineering, and low basal activity(28–30).

Pikromycin (1) is a 14-membered macrolide antibiotic whose carbon skeleton is biosynthesized by a prototypical, modular PKS *(pikAI-pikAIV)* in *Streptomyces venezuelae* ATCC 15439. Its structure is similar to the semisynthetic ketolide antibiotics that show activity against respiratory pathogens resistant to traditional antibiotics(31). This resemblance has triggered researchers to generate pikromycin derivatives through combinatorial biosynthesis(32–35). However, because the modular PKSs of *Streptomyces* are encoded by lengthy genes with high GC content, the efficacy of conventional cloning techniques is significantly hampered. To overcome this, various *in vitro* and *in vivo* CRISPR-Cas9 editing systems as well as allelic exchange methodologies have been developed(36–39). However, the *in vivo* approaches still face challenges, including the cytotoxicity of Cas9, low efficiency of homologous recombination, and the low success rates of editing extensive DNA regions. Riboswitches have improved genome editing efficiency in *Streptomyces* through reducing the cytotoxic effects associated with Cas9 expression; however, their application is still limited to gene deletion(40). Recently, pKCcas9dO CRISPR-Cas9 was developed and used to both delete cytochrome P450 and ferredoxin genes (~ 200–2000 bp) as well as insert kasOp* (~ 100 bp) into the genome of *S. venezuelae* ATCC 15439; however, chromosomal instability during this editing caused the loss of a large genomic region (0.6 Mbp, 6.7% of the genome), inversion and translocation of a large DNA segment, additional copy of a genome fragment, and unexpected loss of genes(41, 42).

In this study, a CRISPR-Cas9-based system was designed to efficiently edit extensive DNA regions within the pikromycin biosynthetic gene cluster of *S. venezuelae*. This system employs a constitutive ermE* promoter and a modified theophylline-inducible riboswitch E* to co-regulate the expression of Cas9. This enhances transformation efficiency by mitigating the cytotoxicity associated with Cas9 while still exerting robust genetic-editing capabilities upon induction. The system also includes the segregationally-unstable pIJ101 replicon to help prevent undesirable genetic rearrangements often encountered when editing the repetitive genes of modular PKSs(43). Using this CRISPR-Cas9 system, we demonstrate the ability to precisely delete or replace large DNA fragments. Seamless, *in vivo* module-swapping was performed within *S. venezuelae to* yield two new pikromycin derivatives that were characterized by NMR and are nearly as active against *Bacillus subtilis* as pikromycin.

## METHODS

### Bacterial strains, plasmids, and media

The bacterial strains and plasmids utilized in this research are outlined in Table S1. *Escherichia coli* DH5α competent cells were employed for standard cloning procedures. *S. venezuelae ATCC* 15439 served as the host strain for the engineering and expression of the hybrid pikromycin derivatives. *B. subtilis* strain 168 was utilized for the microbial susceptibility assessment. *E. coli* cultures were maintained at 37°C on LB agar plates or broths containing the appropriate antibiotics, with liquid cultures incubated on a shaker platform at 200 rpm. Various media, including mannitol soya flour (MS) agar (20 g/L mannitol, 20 g/L soya flour, 20 g/L agar), R5 regeneration medium agar (22 g/L agar, 103 g/L sucrose, 0.25 g/L K_2_SO_4_, 10.12 g/L MgCl_2_·6H_2_O, 10 g/L glucose, 0.1 g/L casamino acid, 5 g/L yeast powder, 5.73 g/L TES, 0.005% KH_2_PO_4_, 0.02M CaCl_2_, 0.3% l-proline, 0.007 N NaOH, and 0.002% trace element solution), SPA medium agar (1 g/L yeast extract, 1 g/L beef extract, 2 g/L tryptone, 10 g/L glucose, trace amount of FeSO4, and 15 g/L agar), AS-1 agar (1 g//L yeast extract, 5 g/L soluble starch, 0.2 g/L l-alanine, 0.2 g/L l-arginine, 0.5 g/L l-asparagine, 2.5 g/L NaCl, 10 g/L Na_2_SO_4_, and 20 g/L agar), TSB medium agar (17 g/L tryptone, 3 g/L soytone, 2.5 g/L dextrose, 5 g/L NaCl, 2.5 g/L K_2_HPO_4_, 15 g/L agar) and fermentation medium (10 g/L glucose, 10 g/L glycerol, 10 g/L polypeptone, 5 g/L meat extract, 5 g/L NaCl, 2 g/L CaCl_2_, 1 g/L yeast extract)(32, 44–46), were used to support the growth and production of pikromycin derivatives. Antibiotics used included apramycin (50 μg/mL), kanamycin (50 μg/mL), chloramphenicol (35 μg/mL), and nalidixic acid (25 μg/mL).

### Construction of non-targeting CRISPR/Cas9 plasmids

In this study, we engineered seven distinct CRISPR-Cas9 expression systems, designated as pMKR02 through pMKR08, through a multistep fabrication process. Initially, we PCR-amplified a DNA fragment, which contained the *Streptomyces-optimized cas9* gene, guide RNA scaffold, apramycin resistance gene, and ColE1 origin of replication, from the pCRISPomyces-2 plasmid using primer set P1/P2(47). This amplified segment was then integrated into the wzc05 plasmid using NdeI and XbaI restriction sites, generating an intermediate construct termed wzc-cas9(48). Subsequently, we further PCR-amplified a DNA fragment from the *wzc-cas9* plasmid using primer set P3/P4 and assembled it with two additional components: the RK2 conjugal transfer origin and the pIJ101 replicon segment, which were amplified using primer set P5/P6 and set P7/P8 individually from pYH7, through Gibson assembly (New England Biolabs, E2611S), resulting in the CRISPR-Cas9 system, pMKR08(49). To develop the theophylline-inducible variants, pMKR02–07, we first synthesized the riboswitch DNA elements by annealing complementary primer sets, P9/P10, P11/P12, P13/P14, P15/P16, P17/P18, and P19/P20 respectively, and then fused them with DNA fragments amplified from pMKR08 using primer sets P21/P22 and P23/P24 via Gibson assembly. Additionally, for analyzing the transformation efficiency of these CRISPR/Cas9 systems, we constructed pMKR01 plasmid, which contains most DNA fragment of pMKR08 except the region of ermE* promoter and *cas9* gene, by NdeI-digestion and self-ligation of the DNA fragments amplified from pMKR08 with primer sets P25/P26. The accuracy of these constructs, pMKR01-pMKR08, was validated through comprehensive whole plasmid sequencing performed by Plasmidsaurus Inc.

### Construction of specific gene-targeting CRISPR/Cas9 plasmids

The targeting constructs were generated through a combination of Golden Gate assembly and traditional restriction enzyme-based cloning. Specifically, the CRISPR-Cas9 spacer inserts were produced by annealing pairs of 24-nucleotide oligonucleotides, which were then individually integrated into the CRISPR-Cas9 plasmids via Golden Gate assembly with the BbsI enzyme. To create CRISPR-Cas9 plasmids containing dual spacer inserts, two individual plasmids were first constructed, each with a unique spacer. The second spacer was then amplified from one of these plasmids using the P27/P28 primer set, digested with XbaI and SpeI, and inserted into the XbaI site of the other CRISPR-Cas9 plasmid. For deleting DNA fragments through HDR, editing templates with 1.5 kb homology arms were amplified from *S. venezuelae* genome and integrated into the XbaI-digested CRISPR-Cas9-derived plasmids carrying the corresponding spacer inserts. Similarly, to swap variant modules into polyketide synthase genes, the target DNA segments were amplified from the organism’s genome, fused with 1.5 kb left and right repair arms using extension PCR, and then inserted into pMKR07-derived plasmids harboring spacer inserts via XbaI/SpeI digestion and ligation. The correct assembly of all plasmids was verified through restriction enzyme diagnostic digestion and whole plasmid sequencing.

### Transformation efficiency assay

To investigate the impact of the CRISPR/Cas9 system on the transformation efficiency of *S. venezuelae* ATCC 15439, a mixture of the strain’s protoplasts and 100 ng of CRISPR/Cas9 DNA was plated in triplicate using a 1:10 serial dilution. The total number of transformants harboring diverse CRISPR/Cas9 systems was estimated at the same dilution level, with the results presented as the mean ± standard deviation from three independent transformation experiments.

### Genetic manipulation of S. venezuelae ATCC15439

The preparation of protoplasts and transformation of *S. venezuelae* ATCC 15439 were performed using established protocols(44). After transformation, individual colonies harboring CRISPR-Cas9 derivative plasmids for genetic modification were randomly selected, re-streaked onto SPA agar plates supplemented with apramycin and 4 mM theophylline, and incubated at 28°C for 3–5 days. These CRISPR-Cas9 engineered bacterial colonies were then analyzed via PCR targeting the genomic region of interest. Colonies exhibiting the desired genetic modifications were plated on antibiotic-free MS agar at 28°C for 3–5 days to facilitate plasmid clearance. The spores were serially diluted and plated on antibiotic-free SPA agar at 28°C for 3 days. Single colonies were re-streaked onto SPA agar plates supplemented with or without apramycin to confirm the plasmid clearance, and the mutants of interest were further verified through PCR amplification, identifying the sequence of amplicons using specific primers by Sanger sequencing, or whole-genome sequencing by Plasmidsaurus Inc (Fig. S23).

### Semiquantitative analysis of GusA activities directly on plates

To investigate the impacts of riboswitch E* and modified riboswitch E* on the expression level of downstream gene, we amplified the DNA fragment from pMKR06 and pMKR07 with P110/P111 and P112/P111 respectively, and fused them with *gusA* gene, which was amplified from pGMGUS (addgene #115678) with primer sets P113/P114 and P115/P114 individually, through Gibson assembly method. The resultant reporter plasmids, pE*-Gus and pmE*-Gus plasmids were introduced into *S. venezuelae by* protoplast-mediated transformation and the transformants were cultured on SPA medium agar plate supplemented with apramycin and theophylline. After 1, 2, and 3 days of incubation, each plate was overlaid with 2 mL of 40 mM 5-bromo-4-chloro-3-indolyl-beta-d-glucuronic acid (X-gluc) solution for visualization of GusA activity (50).

### Construction of complementary plasmids

To further validate the precise editing capabilities of the pMKR07 system, we generated a series of complementary plasmids for heterologous expression. Employing the previously reported methodology(51), we amplified DNA fragments containing partial regions of *pikAI, pikAII, desVIII,* and *desR* utilizing specific primer sets. These fragments were then individually cloned into the wzc02 or pMKBAC02 BAC vectors. Subsequently, we utilized protoplast-mediated transformation to integrate these plasmids into the chromosomal DNA of *S. venezuelae* via homologous recombination. The genomic DNA of the transformants was then prepared, digested, and self-ligated. The ligation mixture was transformed into 10G BAC-optimized electrocompetent cells, and the recombinants were selected on apramycin-containing LB-agar. Next, the resulting plasmids were further analyzed to ensure proper construction through whole plasmid sequencing, and then inserted the attP-intΦC31 DNA fragments. The wzc02-derived plasmid containing the *pikAI* gene was named W1, and the pMKBAC02 derived BAC carrying the whole pikromycin PKS cluster was denoted as BP. Finally, we inserted the *pikAI* promoter region amplified from the *S. venezuelae* genome into the plasmids which respectively contain the *pikAII* and *pikAIII-desVIII* regions, to drive the expression of downstream genes, and the resultant plasmids were named W2 and W3.

### Production, isolation, and analysis of compounds

The strains were cultured for 48 hours in the fermentation medium under conditions of 250 rpm and 28°C for the purification of compounds. Following centrifugation, the broth was extracted twice with ethyl acetate and analyzed using LC-HRMS [6230 TOF LC/MS equipped with a ZORBAX RRHD Eclipse Plus C18 Column (2.1 × 50 mm) with a flow rate of 0.2 mL min^−1^ (solvent A, water with 0.1% formic acid; solvent B, acetonitrile with 0.1% formic acid. 2–100% B for 10 min, 100% B for 2 min), positive mode]. The extract was then purified using a silica gel column. The purified compounds were dissolved in methanol and injected onto an Agilent 6230 TOF LC/MS system connected to a CAPCELL PAK MGIII C18 column (250 mm × 4.6 mm, 5 μm) (Osaka Soda), which was equilibrated with 20% acetonitrile and 0.1% formic acid. Elution was performed using a gradient from 20 to 50% acetonitrile for 20 min at a flow rate of 1 mL/min, and the compounds were observed at 225 nm and collected.

### NMR experiments

NMR spectroscopy was conducted using Varian DirectDrive spectrometers operating at different frequencies at the NMR Facility in the Chemistry Department at the University of Texas at Austin. ^13^C NMR spectra were acquired with proton broadband decoupling. Deuterated solvents served as internal references for the NMR measurements, and chemical shifts are reported in parts per million (ppm) relative to CDCl_3_, with ^1^H NMR referenced to 7.26 ppm and ^13^C NMR referenced to 77.16 ppm.

### Chiral chromatography

Purified compounds were dissolved in methanol and injected onto an Agilent 6230 TOF LC/MS connected to a CHIRALPAK^®^IF-3 Column (Daicel) (4.6 × 250 mm) equilibrated with 25% acetonitrile and 0.1% formic acid. Elution was performed through isocratic flow with the same solvent system. Compounds were observed at 225 nm and by ion count.

### MIC assay

The antibacterial activities of **1**-**4** were evaluated against *B. subtilis* strain 168 according to the Clinical and Laboratory Standards Institute (CLSI) broth microdilution method. Freshly cultured *B. subtilis* cells in Mueller-Hinton medium were diluted 1:500 and used as the inoculum. After incubating the cells for 24 hours at 37°C in the presence of two-fold serial dilutions of the compounds, the minimum inhibitory concentration was determined as the lowest concentration that prevented visible bacterial growth in the wells.

## RESULTS

### Impact of riboswitches on the transformation efficiencies of CRISPR-Cas9 systems

To minimize unintended DNA recombination associated with the widely-used, temperature-sensitive pSG5 replicon in CRISPR-Cas9 tools for *Streptomyces,* we selected the segregationally-unstable pIJ101 replicon(52, 53). As this replicon has been successfully utilized in the design of CRISPR-Cas9 tools for deleting highly-repetitive DNA sequences, pIJ101 was incorporated into the construction of our initial CRISPR-Cas9 plasmid, pMKR08(43). The constitutive ermE* promoter was also utilized to access the Cas9 levels required for effective genome editing. To screen for an appropriate theophylline-responsive riboswitch, versions A, B, C, D, and E* were integrated into pMKR08, yielding pMKR02-pMKR06 (Fig. S1) (54, 55). Moreover, recognizing the influence the spacer region between the Shine-Dalgarno (SD) sequence and the start codon has on Cas9 expression(54, 56, 57), the spacer (5’-CAACAAG-3’) of riboswitch E* in pMKR06 was replaced with a shorter, GC-rich spacer (5’-TCCCAT-3’) to generate a modified riboswitch E* as well as pMKR07. This spacer is from the high-expression *Streptomyces* plasmids, wzc-orfA and wzc-orfL(48), which were inspired by pNG2 (accession KR131849)(58). The plasmid pMKR01, which lacks both the ermE* promoter and *cas9* relative to pMKR08, was also constructed as a control in evaluating the effectiveness of these riboswitches in mitigating Cas9-induced cytotoxicity. SgRNAs targeting both ends of *pikAII* as well as a homologous DNA repair template were introduced into each plasmid, and the recombinant plasmids were introduced into *S. venezuelae* via protoplast-mediated transformation. After a 5-day culture period, high transformation efficiency was observed for pMKR01-pikAII, pMKR02-pikAII, pMKR04-pikAII, pMKR05-pikAII, pMKR06-pikAII, and pMKR07-pikAII, but low transformation efficiency was observed for pMKR03-pikAII and pMKR08-pikAII (Fig. 2). This is presumably due to the relative absence or presence of Cas9. Interestingly, pMKR03-pikAII, which expresses Cas9 under the control of riboswitch B, exhibited the lowest transformation efficiency (about 4%) of the riboswitch-containing systems, suggesting that leaky Cas9 expression caused cell damage, as previously reported (54).

### Efficiency of pikAII deletions by CRISPR-Cas9 systems

The effectiveness of CRISPR-Cas9-mediated genome editing depends on the generation of Cas9-mediated DSBs and the engagement of an intrinsic HDR system. In our CRISPR-Cas9 systems, Cas9 expression is dependent not only on theophylline concentration but also on the activity of the ermE* promoter, which is, in turn, dependent on growth conditions(59, 60). Since intracellular ATP levels and HDR efficiency are known to be correlated in *Streptomyces coelicolor,* genome-editing efficiency may be improved in *S. venezuelae by* modulating cellular energy metabolism(40, 61, 62). Accordingly, we investigated the effects of culture media and theophylline concentrations on the efficiency of our CRISPR-Cas9 systems in *S. venezuelae*. AS-1, MS, TSB, and SPA agar were examined, as each contains a different type or concentration of carbon sources (51, 63). The results from the *pikAII*-deletion experiments revealed that CRISPR-Cas9 editing efficiency increased with theophylline concentration and that the highest efficiencies were from induction on SPA medium agar. Remarkably, pMKR07-pikAII (modified riboswitch E*) enabled an 87.5% genetic-editing success rate for the deletion of *pikAII* when transformants were induced on SPA medium agar with 4 mM theophylline (Table 1; Fig. S2). Under the same conditions, pMKR06-pikAII (riboswitch E*) only provided a 25% success rate. Intriguingly, the observed efficacy was not achieved when transformants harboring the pMKR07 system were cultured on AS-1 medium agar, even with identical theophylline induction. These findings underscore the importance of culture media composition and inducer concentration for CRISPR-Cas9-mediated genetic modifications in *S. venezuelae* and suggest higher Cas9 expression under modified riboswitch E* than riboswitch E* post-induction. To further examine the differential effects of riboswitch E* and modified riboswitch E* on downstream gene expression following induction, two gusA-reporter plasmids, pE*-GusA and pmE*-GusA, were respectively generated from pMKR06 and pMKR07 by replacing *cas9* with *gusA*. Leaky GusA expression was observed from both plasmids after 2 days of culturing without theophylline supplementation. This likely explains the reduced transformation efficiency observed for pMKR06 and pMKR07 compared to pMKR01 (Fig. 2). Furthermore, higher GusA expression was observed for modified riboswitch E* than for riboswitch E* when transformants were cultured on SPA medium agar with 4 mM theophylline for 2 days (Fig. S3). This strongly suggests that the enhanced genetic-editing capability of the pMKR07 system over the pMKR06 system is due to increased Cas9 expression.

### Deletion of large DNA regions by the pMKR07 system

To further evaluate the editing capabilities of the pMKR07 system on large DNA regions, a series of deletion experiments targeting *pikAI* (13 kb), *pikAII* (11 kb), the *pikAIII-desVIII* region (11 kb), and the *pikR2-desR* region (47 kb) was conducted. The findings revealed that, while efficiency decreased when deleting larger DNA fragments, the pMKR07 system remained effective deleting DNA segments up to 47 kb in size (20% efficiency) (Table 2) (Fig. S2; S4-S6). Nanopore sequencing indicated that the genomic DNA sequences of gene-deficient mutants were edited precisely as designed without random recombination events (data not shown). Additionally, complementary plasmids, W1, W2, and W3, and the bacterial artificial chromosome (BAC) BP, respectively carrying *pikAI, pikAII, pikAIII-desVIII,* and *pikR2-resR,* were constructed and introduced into their corresponding genetically-deficient strain. Liquid chromatography/high-resolution mass spectrometry (LC/HRMS) analysis of complemented strain culture extracts confirmed that pikromycin production was restored in each case, substantiating the precise genetic-editing ability of the pMKR07 system (Fig. 3).

### A conservative module swap

**Figures 1:**
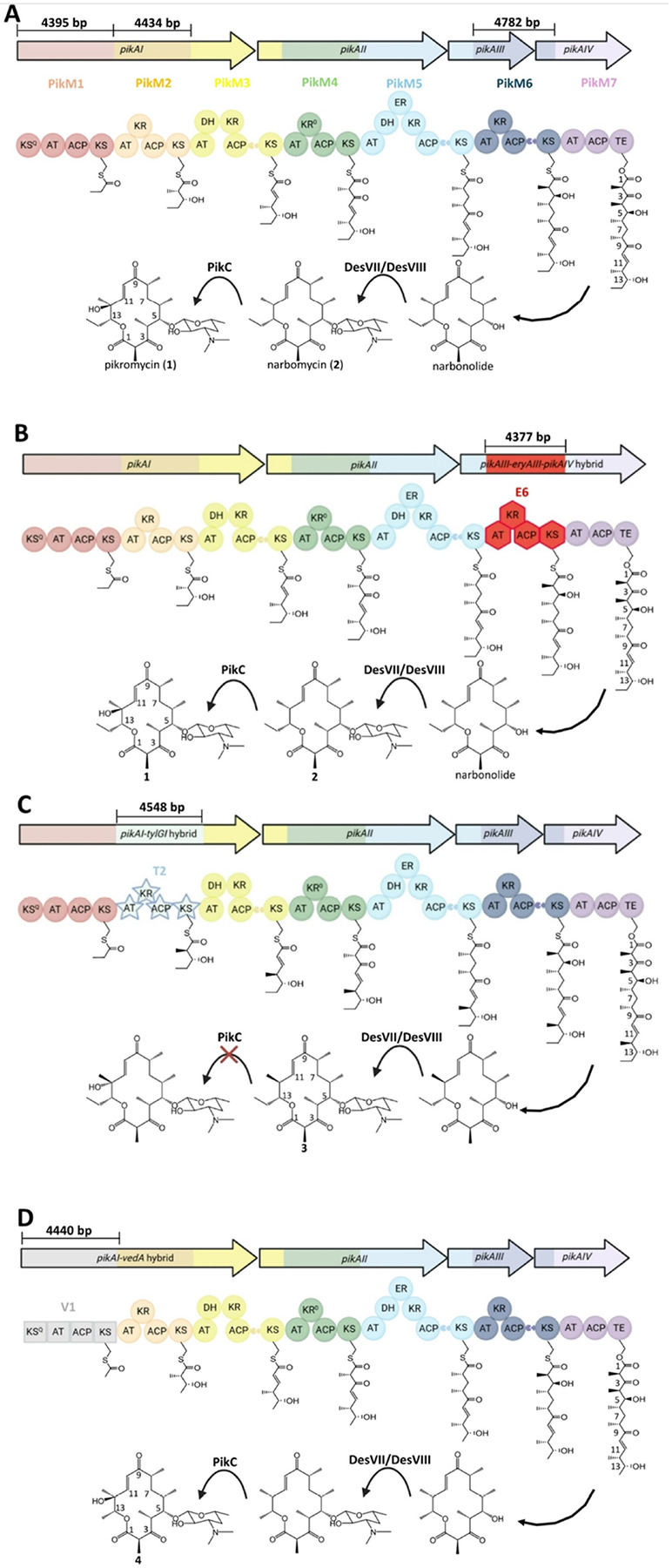
Module-swapping of the pikromycin PKS. A) The 7 modules of the pikromycin PKS (**P1**-**P7**, updated boundaries), are housed by 4 polypeptides that are encoded by *pikAI-pikAIV*. A heptaketide intermediate is cyclized into the 14-membered macrolactone, narbonolide, by the thioesterase (TE) domain. Narbonolide is desosaminylated by DesVII/DesVIII, and narbomycin (2) is hydroxylated by PikC to pikromycin (1). B) The 6^th^ module of the pikromycin PKS was replaced by the 6^th^ module of the erythromycin PKS, and **P6**/**E6**-swapped strain, ZC1, generated pikromycin (1). C) The 2^nd^ module of the pikromycin PKS was replaced by the 2^nd^ module of the tylosin PKS, and **P2**/**T2**-swapped strain, ZC2, generated narbomycin derivative 3. D) The 1^st^ module of the pikromycin PKS was replaced by the 1^st^ module of the venediol PKS, and **P1**/**V2**-swapped strain, ZC3, generated narbomycin derivative **4**. Circles, hexagons, and stars represent enzymatic domains. Docking domains are shown as wedged ends. ACP, acyl carrier protein; KS, ketoacyl synthase; KS^Q^, KS-like decarboxylase; AT, acyltransferase; KR, ketoreductase; KR^0^, KR-like epimerase; DH, hydroxyacyl dehydratase; ER, enoyl reductase; DesVII/DesVIII, the desosaminyltransferase; PikC, cytochrome P450 hydroxylase; **E6**, module 6 of erythromycin PKS; **T2**, module 2 of tylosin PKS; **V1**, module 1 of venediol PKS.

**Figure 2:**
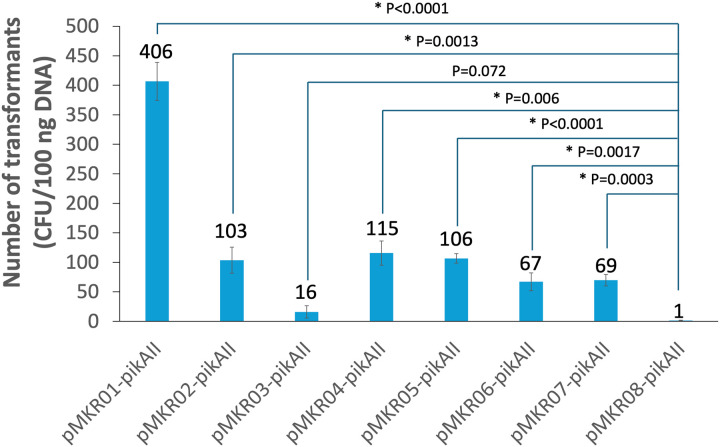
Transformation efficiencies of CRISPR-Cas systems mediating *pikAII* deletion. Each of the plasmids are derived from pMKR08 and contain the same sgRNAs targeting the ends of *pikAII* and homology repair template. pMKR01 does not contain the ermE* promoter or *cas9*. pMKR02-pikAII, pMKR03-pikAII, pMKR04-pikAII, pMKR05-pikAII, pMKR06-pikAII, and pMKR07-pikAII respectively contain theophylline-responsive riboswitches A, B, C, D, E* and a modified E* to modulate Cas9 expression. The average number of transformants per 100 ng plasmid (from 3 independent transformations) are reported with standard deviations. Statistical significance was assessed by a two-tailed unpaired Student’s t-test, and asterisks show statistically significant differences.

**Figure 3:**
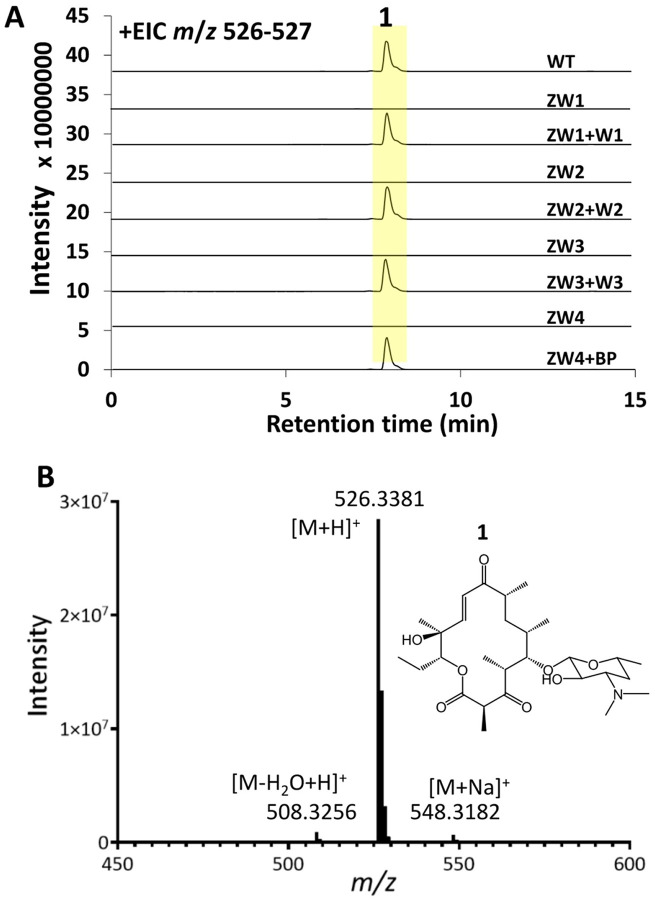
LC/HRMS analysis shows complementation of gene-deleted strains restores pikromycin production. A) Extracted ion chromatograms (EICs) of pikromycin (1) from the culture extracts of *S. venezuelae* ATCC 15439, gene-deleted strains, and complemented strains. WT: *S. venezuelae* ATCC 15439; ZW1: *pikAI-*deleted strain; ZW2: *pikAII*-deleted strain; ZW3: *pikAIII-desVIII-deleted* strain; ZW4: *pikR2-resR-deleted* strain. W1, W2, W3, and BP: Plasmids/BAC carrying *pikAI, pikAII, pikAIII-desVIII,* and *pikR2-resR* for restoring pikromycin production through complementation. B) Electrospray ionization (ESI) mass spectrum of **1** from the *S. venezuelae* ATCC 15439 culture extract. **1**, [M+H]+ observed: 526.3381 *m/z,* expected: 526.3380 *m/z,* 0.19 ppm).

**Figure 4:**
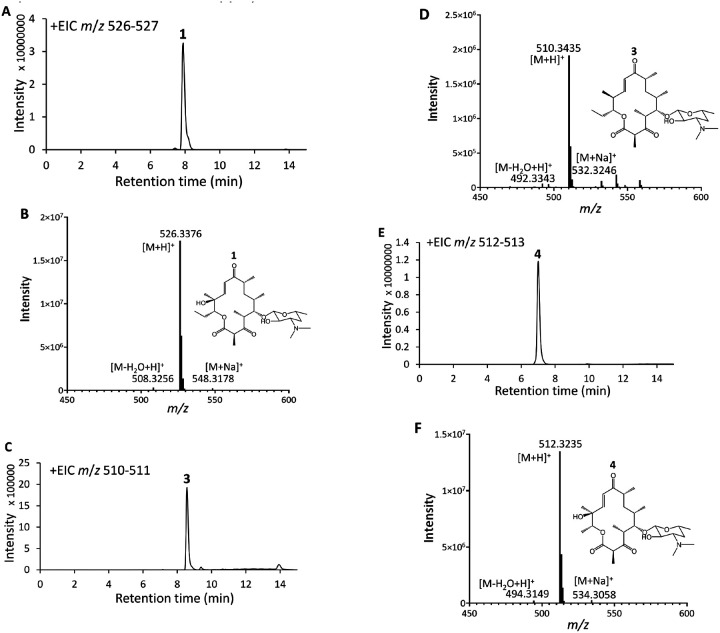
LC/HRMS analyses of **1, 3**, and **4** production by strains harboring module-swapped pikromycin PKSs. A) EIC *(m/z* 526–527, anticipated for **1**) from the culture extract of **P6/E6-**swapped ZC1. B) Mass spectrum of **1** from ZC1. C) EIC *(m/z* 510–511, anticipated for **3**) from the culture extract of **P2/T2-**swapped ZC2. D) Mass spectrum of **3** from ZC2. E) EIC *(m/z* 512–513, anticipated for **4**) from the extract profile of **P1**/**V1**-swapped ZC3 after 2 days of growth. F) ESI mass spectra of **4** detected in the culture of ZC3. **1**, [M+H]+ observed: 526.3376 *m/z,* expected: 526.3380 *m/z,* −0.76 ppm); **3**, [M+H]+ observed: 510.3435 *m/z,* expected: 510.3431 *m/z,* 0.78 ppm); **4**, [M+H]+ observed: 512.3235 *m/z,* expected: 512.3223 *m/z,* 2.34 ppm).

The pikromycin and erythromycin PKSs are highly similar. The 6th module of each synthase (**P6** and **E6**) performs the same chemistry on highly similar polyketide intermediates. However, they are architecturally distinct in that **E6** is housed on a single polypeptide whereas **P6** is housed by two polypeptides connected through docking domain motifs (Fig. 1A and 1B). To investigate the interchangeability of these modules the ability of the pMKR07 system to manipulate large genomic regions, we replaced **P6** with **E6** in the *S. venezuelae* genome (see Methods). Following CRISPR-Cas9 editing and plasmid curing, amplicons generated using the P83/P84 primer set and digested with SacI yielded the anticipated fragments (2.6 and 2.4 kb, rather than 3.8 and 1.6 kb for the wild-type strain) (Fig. S7). Sanger sequencing of these amplicons confirmed the module replacement. The **P6**/**E6**-swapped strain, ZC1, was generated with an editing efficiency of 50%. LC/HRMS analysis of ZC1 culture extract indicated production of **1**. This compound was purified and confirmed to be pikromycin by nuclear magnetic resonance (NMR) (Fig. 4A–B; Fig. S10-S13). The ZC1 strain, with its **P6**/**E6**-swapped pikromycin PKS, produces 1 with a yield of 20 mg/L (80% the wild-type level).

### Module swaps yield two bioactive pikromycin derivatives

**Figure 5:**
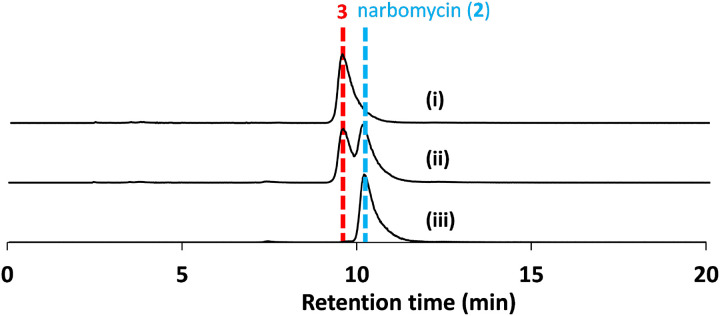
Chromatographic separation between narbomycin and narbomycin epimer **3**. EICs from chiral LC/HRMS analysis of (i) **3**, (ii) mixture of **3** and narbomycin (2), and (iii) **2**.

In the biosyntheses of pikromycin and the related macrolide antibiotic tylosin, the 2nd modules of their respective PKSs (**P2** and **T2**) generate a-methyl, b-hydroxy diketide intermediates; however, their a-methyl groups are oppositely oriented(64) (Fig. 1C). We aimed to reverse the stereochemistry of corresponding methyl group at the C12 position of pikromycin through a **P2**/**T2**-swap and generated the strain ZC2 (see Methods). Following a 2-day fermentation, low levels (0.15 mg/L) of a compound with a molecular weight corresponding to narbomycin, were detected (Fig. 4C and 4D). Compound 3 was isolated and characterized by NMR spectroscopy (Figs. S14-S17) and chiral LC/HRMS (Fig. 5). Its antimicrobial activity was then compared to that of pikromycin (1) and narbomycin (**2**, provided by Takeshi Miyazawa) (65) (Table 3, Fig. S22). MIC measurements show **3** is active against *Bacillus subtilis* strain 168; however, its MIC value is twice that of pikromycin (1) and narbomycin (2).

Structural modification at the C13 position of macrolide antibiotics has yielded derivatives with enhanced activity against resistant bacterial pathogens(66, 67). In contrast to the 1st module of the pikromycin synthase (**P1**) that generates a propionyl starter unit, the 1st module of the venediol PKS (**V1**), also encoded on the *S. venezuelae* ATCC 15439 genome, generates an acetyl starter unit. A swap of these modules could yield a methyl rather than an ethyl substituent at the C13 position of pikromycin. The **P1**/**V1**-swapped strain, ZC3, was engineered, and PCR analyses indicated a Cas9-editing efficiency of 33% (Fig. 1D; Fig. S9, Table 2)(42). A substantial fraction of the mutants remained resistant to apramycin after curing the CRISPR-Cas9 plasmid (pMKR07-P1V1). We hypothesize this is due to the integration of the plasmid into the *S. venezuelae* genome, facilitated by the homologous DNA fragment of the **V1** module carried on the plasmid. After 2 days of culturing, ZC3 produced compound **4** (7.5 mg/L, 30% of **1** from *S. venezuelae* ATCC 15439), whose HRMS and NMR spectra are consistent with the anticipated C13-methyl derivative of pikromycin (Fig. 4E–F; Fig. S18-S21). MIC measurements against *B. subtilis* show that **4** is half as active as pikromycin (Table 3).

## DISCUSSION

Synthetic biologists primarily choose between two approaches when engineering hybrid natural product pathways. The first is to place genes from biosynthetic pathways on vectors, such as plasmids, BACs, or yeast artificial chromosomes (YACs), and express them in heterologous hosts; however, problems often arise due to host incompatibilities, limited precursors, complex regulation, and toxicity(14, 32, 39, 68, 69). The second approach is to directly edit the genomes of native producer strains(37, 70–72). This can maintain the native regulatory mechanisms and metabolic infrastructure of the host but is more technically challenging. To accelerate the genome modification of streptomycetes, researchers have developed various strategies, including using alternative endonucleases *(e.g.,* Cas3, Cas12a) and decoupling the transformation and editing steps through inducible Cas9 expression(73–76). Despite these advances, modifying large DNA regions within genomes has remained a challenge(38).

In this work, 6 theophylline-responsive riboswitches (A-E* and modified E*) were employed to minimize cytotoxicity from Cas9 during transformation and maximize the frequency of genome modification after Cas9 induction (Fig. S1)(54). Riboswitch B resulted in the lowest transformation efficiency, suggesting that Cas9 expression was leaky and cytotoxic. No *pikAII*-deletion mutants were observed when employing pMKR03-pikAII under various culture conditions, either in the presence and absence of theophylline. As previous studies with riboswitch B in *S. coelicolor* observed leaky expression, leaky Cas9 expression may have impeded cell growth (77). Although plasmids harboring riboswitches A, C, and D show high transformation efficiencies, they also did not yield *pikAII*-deletion mutants, even after culturing with various media supplemented with theophylline. From research on theophylline-responsive riboswitches in *S. coelicolor*(55), we hypothesize post-induction Cas9 expression levels were insufficient. From the results of the GusA reporter assay (Fig. S3), we hypothesize that higher genetic-editing capability correlates with post-induction Cas9 expression levels. Furthermore, our tests on the effects of various culture media on genetic editing revealed higher editing efficiency when transformants were cultured and induced on nutrition-rich medium agar plates that promoted faster growth. Bacterial aerobic respiration generates ATP through the metabolic processes of glycolysis and oxidative phosphorylation. The available carbon sources, especially glucose as the primary energy substrate for cellular metabolism, significantly influence ATP levels(62, 78, 79). The pMKR07 system demonstrated the highest efficiency deleting *pikAII* when transformants were cultured and induced on SPA medium agar, which has a higher glucose concentration than TSB or MS medium agar. These results show that genome editing is influenced by Cas9 levels and nutrient metabolism. The pMKR07 system reliably performed 4–5 kb swaps, enabling the **P6**/**E6**, **P2**/**T2**, and **P1**/**V1** replacements with success rates of 50%, 37.5%, and 33%, respectively (Table 2). Although these rates are lower than those observed when knocking out *pikAI* (13 kb, 87.5%), *pikAII* (11 kb, 87.5%), and *pikAIII-desVIII* (11 kb, 100%), they surpass the efficiency of deleting the entire pikromycin biosynthetic gene cluster (47 kb, 20%). HDR-mediated modifications of large DNA regions are known to occur at lower rates than HDR-mediated small deletions, insertions, and substitutions(80).

The yield of pikromycin (1) from the **P6**/**E6**-swapped ZC1 is 80% that of wild-type. As studies indicate *pikAIV* **is** co-transcribed with *pikAIII* (Fig. 1B), the **P6**/**E6** swap may not affect the level of mRNA encoding this region of the synthase(81). However, it does result in the production of a 308 kDa hybrid polypeptide rather than PikAIII (164 kDa) and PikAIV (142 kDa) (Fig. 1B). Since transcript length is a key determinant of translation efficiency, the hybrid polypeptide may be present at decreased levels(82, 83). The relative decrease in pikromycin titer could also be related to suboptimal interactions between **E6** and the rest of the pikromycin PKS or the intermediates on which it operates, although a recent study observed similar activities from **P6** and **E6** within engineered PKSs(11, 84).

Module-swapping with the pMKR07 system enabled strains ZC2 and ZC3 to generate new macrolide antibiotics. **P2**/**T2**-swapped ZC2 produces low levels of **3**, the C12-epimer of narbomycin. LC/HRMS analysis of the ZC2 culture extract does not show any compound with the same *m/z* **as** pikromycin (1). Thus, likely due to the orientation of its C12 methyl group, **3** is not a substrate for PikC, which naturally oxidizes the C12 position of narbomycin(85). The low titer of **3** could be a result of its oppositely-oriented C12 methyl group causing suboptimal interactions between intermediates and downstream PKS enzymes, such as the triketide intermediate with the P3 KS or the heptaketide intermediate with the thioesterase (TE)(16, 86). **P1**/**V1**-swapped ZC3 produces pikromycin derivative **4**, which contains a methyl rather than an ethyl group at C13, at a level similar to that of 1 from *S. venezuelae* ATCC 15439. Apparently, the intermediates of the **P1**/**V1**-swapped pikromycin PKS, which are one methylene group shorter than those of the native synthase, are well tolerated by the hybrid PKS. Also, the narbonolide derivatives generated by the **P2**/**T2** and **P1**/**V1**-swapped PKSs are desosaminylated by DesVII/DesVIII, known to be relatively promiscuous, as these derivatives were not observed from the LC/HRMS analyses of the culture extracts(87).

The new macrolide antibiotics **3** and **4** show bioactivities comparable to the natural ketolides pikromycin (1) and narbomycin (2). As these compounds possess alterations to the region that is converted to a carbamate, carbonate, or lactone in semisynthetic ketolides (e.g. telithromycin), they may be of interest to medicinal chemists(88, 89). Modifications at the C13 position of macrolide antibiotics, such as clarithromycin, can improve their bioactivities through enhanced hydrophobic interactions with the ribosome(67). It may be possible to increase the size and lipophilicity of the C13 substituent by replacing **P1** with the first modules of other synthases, such as the borrelidin or rapamycin PKS(90) (91).

## CONCLUSIONS

Developing the pMKR07 CRISPR-Cas9 system with a segregationally-unstable origin of replication and modified theophylline-responsive riboswitch as well as employing it in optimized growth conditions enabled the efficient and precise editing of large DNA regions within *S. venezuelae* ATCC 15439. These findings open opportunities for developing similar systems for editing the genomes of other streptomycetes. The ability to perform module-swapping of the pikromycin PKS and related assembly lines within their native hosts will not only enable scientists to learn more about assembly line logic and engineering but also facilitate the generation of new medicines.

## Figures and Tables

**Table 1.** Optimizing the deletion of *pikAII* through varying riboswitches, theophylline concentration, and media.

	AS-1			MS			TSB			SPA		
TheophyIIine (mM)	0	2	4	0	2	4	0	2	4	0	2	4
pMKR02-pikAII (riboswitch A)	0/8	0/8	0/8	0/3	0/8	0/8	0/8	0/8	0/8	0/8	0/8	0/8
pMKR03-pihAII (riboswitch B)	0/8	0/8	0/8	0/8	0/8	0/8	0/8	0/8	0/8	0/8	0/8	0/8
pMKR04-pikAII (riboswitch C)	0/8	0/8	0/8	0/8	0/8	0/8	0/8	0/8	0/8	0/8	0/8	0/8
pMKR05-pikAII (riboswitch D)	0/8	0/8	0/8	0/8	0/8	0/8	0/8	0/8	0/8	0/8	0/8	0/8
pMKRC6-pikAII (riboswitch E^a^)	0/8	0/8	0/8	0/8	0/8	1/3	0/8	0/8	1/8	0/8	2/8	2/8
pMKRC7-pikAII (modified riboswitch E^a^)	0/8	0/8	0/8	0/8	1/8	3/8	0/8	0/8	1/8	0/8	3/8	7/8
	Results^a^											

Note: AS-1, MS, TSB, and SPA are the media used for induction.

a Number of correctly engineered transformants/total number of transformants screened.

**Table 2:** Genome-editing results employing the pMKR07 system in *S. venezuelae*.

Plasmid	Function	Edited region (bp)	Result^a^
pMKR07-*pikAI*	delet i ng *pikAI* gene	12,640	7/8
pMKR07-*pikAII*	delet i ng *pikAII* gene	10,678	7/8
pMKR07-*pikAIII*	deleting *pikAIII-desVIII* region	10,596	8/8
pMKR07-*pik*	deleting *pikR2-desR* region	47,022	3/15
pMKR07-P6E6	swapping module 6 of pikromycin PKS for module 6 of erythromycin PKS	replacing 4,782 bp DNA with 4,377 bp DNA	4/8
pMKR07-P2T2	swapping module 2 of pikromycin PKS for module 2 of tylosin PKS	replacing 4,434 bp DNA with 4,548 bp DNA	3/8
pMKR07-P1V1	swapping module 1 of pikromycin PKS for module 1 of venediol PKS	replacing 4,395 bp DNA with 4,440 bp DNA	2/6

Note:

a Number of correctly engineered transformants/total number of transformants screened.

**Table 3:** MIC values for macrolides 1–4

Compounds^a^				
Bacteria	pikromycin (1)	narbomycin (2)	**3**	**4**
*B. subtilis* strain 168	12.5 μM	12.5 μM	25 μM	25 μM

a MIC is defined as the lowest concentration of analyte with no visible growth.

## Data Availability

All data generated or analysed during this study are included in this published article and its supplementary information file.

## Abbreviations

PKS: polyketide synthase
AT: acyltransferase (AT)
ACP: acyl carrier protein (ACP)
KS: ketosynthase (KS)
KR: ketoreductase (KR)
DH: dehydratase (DH)
ER: enoylreductase (ER)
TE: thioesterase (TE)
KS^Q^: KS-like decarboxylase
KR^0^: KR-like epimerase
CRISPR: clustered regularly interspaced short palindromic repeats
Cas9: CRISPR-associated protein 9
sgRNA: single-guide RNA (sgRNA)
DSB: double-strand DNA break
NHEJ: non-homologous end-joining
P1-P7: the 7 modules of the pikromycin PKS
E6: the 6^th^ module of the erythromycin PKS
T2: the 2^nd^ module of the tylosin PKS
V1: 1^st^ module of the venediol PKS
DesVII/DesVIII: the desosaminyltransferase in the pikromycin pathway
PikC: the cytochrome P450 hydroxylase in the pikromycin pathway
X-gluc: 5-bromo-4-chloro-3-indolyl-beta-d-glucuronic acid
SD: Shine-Dalgarno
NMR: nuclear magnetic resonance
PCR: polymerase chain reaction
ATP: adenosine 5’-triphosphate
LC/HRMS: liquid chromatography/high-resolution mass spectrometry
